# Changes induced by dietary energy intake and divergent selection for muscle fat content in rainbow trout (*Oncorhynchus mykiss*), assessed by transcriptome and proteome analysis of the liver

**DOI:** 10.1186/1471-2164-9-506

**Published:** 2008-10-29

**Authors:** Catherine-Ines Kolditz, Gilles Paboeuf, Maïena Borthaire, Diane Esquerré, Magali SanCristobal, Florence Lefèvre, Françoise Médale

**Affiliations:** 1 INRA, UMR 1067 Nutrition Aquaculture & Genomics – Pôle d'Hydrobiologie, F-64310 Saint-Pée-sur-Nivelle, France; 2 INRA, UR 1037 SCRIBE – Campus de Beaulieu – F-35042 Rennes Cedex, France; 3 INRA, DGA, UMR 314, Laboratoire de Radiobiologie et d'Etude du Génome, CRB GADIE, F-78350 Jouy en Josas, France; 4 INRA, UMR 444, Laboratoire de Génétique Cellulaire, F-31326 Castanet-Tolosan, France

## Abstract

**Background:**

Growing interest is turned to fat storage levels and allocation within body compartments, due to their impact on human health and quality properties of farm animals. Energy intake and genetic background are major determinants of fattening in most animals, including humans. Previous studies have evidenced that fat deposition depends upon balance between various metabolic pathways. Using divergent selection, we obtained rainbow trout with differences in fat allocation between visceral adipose tissue and muscle, and no change in overall body fat content. Transcriptome and proteome analysis were applied to characterize the molecular changes occurring between these two lines when fed a low or a high energy diet. We focused on the liver, center of intermediary metabolism and the main site for lipogenesis in fish, as in humans and most avian species.

**Results:**

The proteome and transcriptome analyses provided concordant results. The main changes induced by the dietary treatment were observed in lipid metabolism. The level of transcripts and proteins involved in intracellular lipid transport, fatty acid biosynthesis and anti-oxidant metabolism were lower with the lipid rich diet. In addition, genes and proteins involved in amino-acid catabolism and proteolysis were also under expressed with this diet. The major changes related to the selection effect were observed in levels of transcripts and proteins involved in amino-acid catabolism and proteolysis that were higher in the fat muscle line than in the lean muscle line.

**Conclusion:**

The present study led to the identification of novel genes and proteins that responded to long term feeding with a high energy/high fat diet. Although muscle was the direct target, the selection procedure applied significantly affected hepatic metabolism, particularly protein and amino acid derivative metabolism. Interestingly, the selection procedure and the dietary treatment used to increase muscle fat content exerted opposite effects on the expression of the liver genes and proteins, with little interaction between the two factors. Some of the molecules we identified could be used as markers to prevent excess muscle fat accumulation.

## Background

The levels of fat storage and allocation within body compartments have become the focus of critical interest during the last few years, due to their impact on human health [1] and meat quality of farm animals [2,3]. Dietary manipulation and genetic selection constitute the two ways to manage body fat content in farm animals. Energy intake and genetic factors also have a major influence on fattening in humans [4].

Storage of triglycerides in the different body compartments depends on the availability of plasma lipids originating from either the diet or lipogenesis. In fish, human and most avian species, lipogenesis mainly takes place in the liver [5,6] and is negligible in muscle [7,8]. The liver has a central role in metabolic homeostasis and in coordinating body metabolism in response to dietary conditions. An increase in dietary lipid generally leads to modification of lipid metabolism in most animals, with inhibition of lipogenic enzymes [9,6,10], and stimulation of fatty acid oxidation [11], especially when the dietary fatty acids are provided as polyunsaturated fatty-acids (PUFAs) [12-14]. However, most of the studies investigating the hepatic metabolic changes induced by long term feeding a high fat diet focused on lipid metabolism.

Body fat distribution is clearly a heritable trait [15]. In farm animals, genetic selection has been used to manage fat content of target body compartments [16,17], but it generally leads to changes in whole body fat content. In humans, studies comparing groups with different fat distribution patterns have highlighted differences in postprandial plasma metabolites [18-20], suggesting differences in fat, glucose and protein metabolism. However, the physiological mechanisms responsible for these differences have not been described to date.

Using divergent selection on muscle fat content in rainbow trout, we obtained animals that were characterized by differences in fat allocation between visceral adipose tissue and muscle, with no change in overall body fat content between lines. We then decided to characterize the differences in metabolic changes occurring between these two lines when fed a low or a high energy diet. We focused on the liver, since this organ is the center of intermediary metabolism and main site for lipogenesis in fish [5]. The aims of the study were 1) to assess the overall changes in gene and protein expression induced in the rainbow trout liver by long term feeding of a high energy/high fat diet, 2) to identify the differences in gene and protein expression profiles induced in the liver as a consequence of the selection for muscle fattening, and 3) to evaluate to what extent the two factors used to modulate muscle fat content may have interacted on the different metabolism-related genes and proteins. This was achieved through two complementary approaches at the transcriptomic and proteomic levels, using microarray and bidimensional electrophoresis.

## Results

### Growth, biometry and biochemical parameters (Table 1)

**Table 1:** Fish growth, morphological and biochemical parameters, and whole body and muscle lipid content

	**L line**		**F line**		p-values		
					
Diet	**LE**	**HE**	**LE**	**HE**	Diet	Line	Line*Diet
Daily Growth index (% day^-1^)							
	2.57 ± 0.04	2.67 ± 0.05	2.37 ± 0.01	2.52 ± 0.03	*p = 0.011*	*p = 0.043*	p = 0.64
Final body weight (g)							
	73.0 ± 2.9	92.4 ± 2.9	57.8 ± 2.0	77.4 ± 2.5	*p = 10*^-4^	*p = 10*^-4^	p = 0.66
Whole body lipid content (% WW)							
	9.5 ± 0.2	15.3 ± 1.1	10.8 ± 0.5	15.2 ± 1.0	*p = 10*^-4^	p = 0.27	p = 0.16
HSI (%)							
	1.3 ± 0.2	1.2 ± 0.2	1.3 ± 0.2	1.3 ± 0.3	p = 0.14	p = 0.99	p = 0.33
VSI (%)							
	8.3 ± 0.8^c^	12.4 ± 1.3^a^	7.7 ± 0.9^d^	11.0 ± 1.9^b^	*p = 10*^-4^	*p = 10*^-4^	*p = 0.003*
Muscle lipid content (% WW)							
	4.3 ± 0.8^c^	6.4 ± 1.2^b^	6.3 ± 1.2^b^	10.1 ± 2.3^a^	*p < 10*^-4^	*p < 10*^-4^	*p = 0.003*
Plasma triglycerides (g/l)							
	5.52 ± 0.77	4.38 ± 1.31	5.74 ± 0.84	4.83 ± 1.31	*p < 10*^-4^	*p = 0.03*	p = 0.47
Plasma glucose (mg/l)							
	81.42 ± 6.68	80.90 ± 5.61	81.34 ± 6.16	81.01 ± 5.71	p = 0.61	p = 0.97	p = 0.91

% WW, percentage of wet weight. Values are expressed as means ± SD (n = 57 individuals in all groups, except for muscle lipid content, for which n = 30); HIS, hepato-somatic index; VSI, Viscero-somatic index; p-values are presented in italics when the differences are significant (p < 0.05, MANOVA 2 factors). Means with different superscript letters are significantly different (p < 0.05, ANOVA).

The higher energy content of the HE diet enhanced growth rate (p = 10^-4^) of fish irrespective of the line. At the end of the feeding trial, fish of the lean muscle line (L) showed higher whole body weight (p = 10^-4^) compared to those of the fat muscle line (F). Whole body lipid content was increased in fish fed the HE diet compared to those fed the low energy diet (LE), and was similar for the two lines fed the same diet. Muscle lipid content was higher in fish fed the HE diet (p < 10^-4^) and in the F line (p < 10^-4^). Viscero-somatic index (VSI), used as an indicator of fat deposition as visceral adipose tissue, was increased in fish fed the HE diet (p = 10^-4^) and in fish from the L line (p = 10^-4^). Plasma triglyceride levels were higher in fish fed the LE diet (p = 10^-4^) and in fish of the F line (p = 0.02). Glycemia was not significantly different between diets (p = 0.61) or lines (p = 0.97) 24 hours after the meal.

### Microarray data analysis

#### Hepatic transcripts differentially expressed between the dietary treatments

The results derived from ANOVA (p < 0.01) and SAM (FDR < 0.15) analysis for global dietary effects (whatever the lines) on hepatic gene expression are summarized in Table 2. A comparison of the two lists of genes generated by these two analytical methods [see additional file 1] revealed that 106 genes were significant according to both statistical analyses. With regard to the ontology of these 106 clones, 83 were attributed a biological function (Table 2). Our study was focused more particularly on genes involved in the metabolic process since it was the largest category represented (44.6% of the transcripts that had a biological function). Lipid metabolism was the metabolic pathway that contained the majority of the differentially expressed transcripts (40.5%), with, in particular, genes encoding for proteins involved in lipid transport (acyl-CoA binding protein [ACBP], heart-type fatty acid-binding protein [H-FABP]), fatty acid desaturation (delta-6-desaturase [Δ6-FAD]) and cholesterol/steroid metabolism (cholesteryl ester transfer protein [CETP]), down-regulated in trout from both lines fed the HE diet (Table 3). There were two different transcripts corresponding to the H-FABP protein (of ~800 bp and ~600 bp) that shared 88% of sequence similarity. Their deduced amino acid sequences contained 94% similar residues, suggesting that these two transcripts may be different isoforms of H-FABP.

**Table 2:** Overview of transcripts in livers of rainbow trout significantly affected by the dietary treatment (HE vs LE diet)^1^

**Statistical analysis**	**ANOVA **(p < 0.01)	**SAM **FDR ≤ 0.15	**ANOVA∩SAM**
**Total**	**165**	**111**	**106**
**HE > LE**	37	8	8
**HE < LE**	128	103	98

**Number of transcripts with known biological function**	**132**	**87**	**83**
**Biological function**			
**Metabolism**	**52 (39.4%)**	**38 (43.7%)**	**37 (44.6%)**
*Lipid*	*19 (14.4%)*	*15 (17.2%)*	*15 (18.1%)*
*Energy*	*7 (5.3%)*	*5 (5.7%)*	*5 (6.0%)*
*Carbohydrate*	*1 (< 1%)*	*1 (1.1%)*	*1 (1.2%))*
*Amino acid and derivative*	*5 (3.8%)*	*3 (3.4%)*	*3 (3.6%)*
*Protein folding/synthesis/breakdown*	*11 (8.3%)*	*6 (6.9%)*	*6 (7.2%)*
*Xenobiotic and oxidant metabolism*	*5 (3.8%)*	*4 (4.6%)*	*4 (4.8%)*
*Purine and pyrimidine*	*4 (3%)*	*4 (4.6%)*	*3 (3.6%)*
Transcription/translation	21 (15.9%)	13 (15.1%)	12 (14.5%)
Cell cycle	13 (9.8%)	6 (6.9%)	6 (7.2%)
Trafficking	9 (6.8%)	6 (6.9%)	6 (7.2%)
Signal transduction	8 (6.1%)	7 (8.0%)	7 (8.4%)
Extracellular matrix and structural components	6 (4.5%)	5 (5.7%)	5 (6%)
Immune and stress response	6 (4.5%)	5 (5.7%)	4 (4.8%)
Others	17 (12.9%)	7 (8.0%)	6 (7.2%)

^1 ^According to ANOVA (p < 0.01), SAM analysis (FDR < 0.15) and both statistical methods

**Table 3:** Metabolism-related hepatic transcripts exhibiting differential expression between the two dietary groups (HE vs LE)^1^

***Biological function***		HE/LE ratio		ANOVA	SAM
				
GenBank Acc. N°	Best-hit Swiss-Prot description	L line	F line	P-value	FDR cut off
***Lipid metabolism (40.5%)***					
CA344881	Trifunctional enzyme subunit alpha, mitochondrial precursor	-1.3	-1.4	< 5.10^-4^	0
BX885839	Acetyl-CoA acetyltransferase, cytosolic (ACAT2)	-1.5	-2.1	< 10^-3^	0
BX861803	Phosphatidylinositol-glycan biosynthesis class F protein (PIG-F)	-1.3	-1.2	0.003	0.1
BX080468	Ectonucleotide pyrophosphatase/phosphodiesterase 7 precursor	-1.3	-1.1	0.004	0.15
***Fatty acid desaturation ****(2.7%)*					
CA371783	putative delta-6 fatty acid desaturase (Δ6-FAD)	-1.9	-2.0	0.005	0.05
***Lipid transport ****(10.8%)*					
CU069821	Acetyl coenzyme A binding protein (ACBP)	-2.1	-1.9	< 5.10^-5^	0
BX078901	Vitellogenin precursor (VTG)	-1.2	-1.4	< 10^-3^	0.05
CU069693	Heart-type fatty acid binding protein (H-FABP)	-1.4	-1.5	< 10^-3^	0
BX298066	Heart-type fatty acid binding protein (H-FABP)	-1.6	-1.5	0.004	0.05
***Cholesterol metabolism ****(16.2%)*					
BX875391	Probable ergosterol biosynthetic protein 28 (ERG28)	-1.8	-2.3	< 5.10^-6^	0
CU069450	24-dehydrocholesterol reductase precursor (DHC24)	-1.8	-2.7	< 5.10^-4^	0
CA344888	Retinol dehydrogenase 12 (RDH12)	-1.2	-2.0	0.003	0.05
CA382526	Orphan nuclear receptor NR1D2 (Rev-erbβ)	-1.2	-1.2	0.004	0.15
CA377380	C-4 methylsterol oxidase (ERG25)	-1.2	1.5	0.006	0.1
CU069541	Cholesteryl ester transfer protein precursor (CETP)	-1.4	-1.2	0.006	0.15
***Generation of precursor metabolites and energy (13.5%)***					
BX084640	6-phosphogluconate dehydrogenase, decarboxylating (6-PGD)	-1.4	-2.2	< 5.10^-4^	0
BX080843	Ubiquinol-cytochrome c reductase complex 9.5 kDa protein	-1.4	-1.3	< 5.10^-4^	0
BX886412	ATP synthase subunit alpha, mitochondrial precursor (ATPA)	-1.1	-1.4	0.001	0.1
CA377924	Vacuolar ATP synthase subunit δ (V-ATPase δ subunit)	-1.2	-1.4	0.002	0.05
CU067427	Pyruvate carboxylase, mitochondrial precursor	-1.2	-1.1	0.008	0.15
***Carbohydrate metabolism (2.7%)***					
CA383037	Solute carrier family 2, facilitated glucose transporter member 11	-1.7	-1.6	< 10^-4^	0
***Amino-acid derivative metabolism (8.1%)***					
CU068986	Acetylcholinesterase precursor (EC 3.1.1.7) (AChE)	-1.7	-1.7	< 5.10^-4^	0
CU070780	D-3-phosphoglycerate dehydrogenase (3-PGDH)	-1.4	-1.2	0.002	0.05
CU067302	Cystathionine gamma-lyase (CGL)	-1.4	-1.0	0.008	0.15
***Protein folding/synthesis/breakdown (16.2%)***					
CU072931	Tripeptidyl-peptidase 2	-1.3	-1.7	< 10^-4^	0
CA362332	Ubiquitin-conjugating enzyme E2 E1 (UB2E1)	-1.3	-1.3	0.002	0.15
CU068585	STIP1 homology and U box-containing protein 1	-1.4	-1.5	0.003	0.05
BX307921	McKusick-Kaufman/Bardet-Biedl syndromes putative chaperonin	-1.2	-1.4	0.005	0.15
CA342952	Proteasome subunit beta type 7 precursor (PSB7)	-1.2	-1.1	0.01	0.15
CU066852	F-box/WD repeat protein 2	-1.4	-1.1	0.03	0.1
***Xenobiotic and oxidant metabolism (10.8%)***					
CU070243	Peptide methionine sulfoxide reductase	-1.3	-1.3	10^-3^	0.05
CU071592	Cytochrome P450 2J2 (Arachidonic acid epoxygenase)	1.9	2.2	0.003	0.1
BX081745	Matrix metalloproteinase-16 precursor	1.9	1.6	0.003	0.15
BX308633	Stress-activated protein kinase 3 (MK12)	-1.2	-1.3	0.008	0.15
***Purine metabolism (8.1%)***					
CU073027	Ectonucleoside triphosphate diphosphohydrolase 1	-1.3	-1.1	< 10^-3^	0.05
CA363765	Adenylate kinase isoenzyme 2, mitochondrial	-1.3	-1.2	< 10^-4^	0.05
CA371563	Ribonucleoside-diphosphate reductase large subunit	2.6	3.9	0.001	0.05

^1 ^According to both ANOVA (p < 0.01) and SAM (FDR < 0.15) analysis

Several clones corresponding to enzymes involved in generation of precursor metabolites and energy (6-phosphogluconate dehydrogenase [6-PGD], pyruvate carboxylase [PC] and subunits of the respiratory chain complexes), amino acid metabolism (D-3-phosphoglycerate dehydrogenase [3-PGDH], cystathionine γ-lyase [CGL] and acetylcholinesterase precursor [AChE]) and proteolysis were also found to be significantly reduced in the livers of fish fed the HE diet compared to those fed the LE diet.

#### Hepatic transcripts affected by the selection procedure

The results derived from ANOVA (p < 0.01) and SAM (FDR < 0.15) analysis for global genetic selection effects (whatever the diet) on hepatic gene expression are summarized in Table 4. [For a complete list of genes identified as significantly different between the two lines according to each statistical approach see Additional file 2]. Seventy transcripts were found to be significant by both statistical methods. Transcripts involved in metabolic pathways accounted for 46% of the transcripts that had received a biological function (Table 4). Only two of these transcripts were involved in lipid metabolism (ceramide kinase 1 and a nonspecific lipid-transfer protein) (Table 5). A greater proportion of differential transcripts encoded for proteins involved in xenobiotic and oxidant metabolism (22.2%), generation of precursors and energy (6-PGDH, malate dehydrogenase [MDH], ubiquinol cytochrome c reductase), amino-acid metabolism (transcripts encoding for two aspartate aminotransferases [GOT], a betaine-homocysteine S-methyltransferase [BHMT] and a 4-aminobutyrate aminotransferase) and proteolysis (proteasome and protein ubiquitinylation components). All were expressed at higher levels in the F line than in the L line (Table 5).

**Table 4:** Overview of hepatic transcripts exhibiting differential expression between the two genotypes (F vs L)^1^

**Statistical analysis**	**ANOVA **(p < 0.01)	**SAM **FDR ≤ 0.15	**ANOVA∩SAM**
**Total**	**154**	**77**	**70**
**F > L**	90	65	58
**F < L**	64	12	12

**Transcripts with known biological function**	**127**	**64**	**58**

**Biological function**			
**Metabolism**	**47 (37.0%)**	**30 (46.7%)**	**27 (46.6%)**
*Lipid*	*(4.67%)*	*2 (3.1%)*	*2 (3.4%)*
*Precursor metabolites and energy*	*7 (5.5%)*	*6 (9.4%)*	*5 (8.6%)*
*Carbohydrate*	*2 (1.6%)*	*1 (1.6%)*	*1 (1.7%)*
*Amino acid and derivative*	*9 (7.1%)*	*6 (9.4%)*	*5 (8.6%)*
*Protein folding/synthesis/breakdown*	*11 (8.7%)*	*6 (9.4%)*	*5 (8.6%)*
*Xenobiotic and oxidant*	*7 (5.5%)*	*6 (9.4%)*	*6 (10.3%)*
*Purine and pyrimidine*	*3 (2.4%)*	*3 (4.7%)*	*2 (3.4%)*
*Iron*	*2 (1.6%)*	*1 (1.6%)*	*1 (1.7%)*
Transcription/translation	19 (15.0%)	3 (4.7%)	2 (3.4%)
Immune response	7 (5.5%)	5 (7.8%)	5 (8.6%)
Trafficking	11 (8.7%)	5 (7.8%)	5 (8.6%)
Signal transduction	7 (5.5%)	3 (4.7%)	3 (5.2%)
Cell cycle	9 (7.1%)	5 (7.8%)	4 (6.9%)
Extracellular matrix and structural components	9 (7.1%)	3 (4.7%)	2 (3.4%)
Others	18 (14.2%)	10 (15.6%)	10 (17.2%)

1 According to ANOVA (p < 0.01), SAM analysis (FDR < 0.15) and both statistical methods

**Table 5:** Hepatic transcripts exhibiting differential expression between the two genotypes (F vs L)^1^

***Biological function***		F/L ratio		ANOVA	SAM
				
GenBank Acc. N°	Best-hit Swiss-Prot description	LE diet	HE diet	P-value	FDR cut off
***Lipid metabolism (7.4%)***					
BX857103	Ceramide kinase	-1.6	-2.8	< 10^-3^	0.1
CA376046	Nonspecific lipid-transfer protein (NLTP)	1.4	1.1	10^-3^	0.1
***Generation of precursor metabolites and energy (18.5%)***					
CA366638	10-formyltetrahydrofolate dehydrogenase	1.8	1.5	< 5.10^-4^	0
CA342644	6-phosphogluconate dehydrogenase (6-PGDH)	2.0	1.3	0.002	0.15
BX860760	10-formyltetrahydrofolate dehydrogenase	2.0	1.9	0.003	0
CA351158	Malate dehydrogenase, cytoplasmic (MDH)	1.4	1.2	0.003	0.1
BX301878	Ubiquinol-cytochrome c reductase iron-sulfur subunit	1.6	1.4	0.006	0.1
***Carbohydrate metabolism (3.7%)***					
BX306300	Protein phosphatase 1 regulatory subunit 3D	1.3	1.5	0.008	0.15
***Amino-acid derivative metabolism (18.5%)***					
CA343008	Betaine-homocysteine S-methyltransferase (BHMT)	1.5	1.2	< 5.10^-4^	0
BX076291	Aspartate aminotransferase, mitochondrial (GOT2)	1.6	1.2	10^-3^	0.1
CA353510	Aspartate aminotransferase, mitochondrial (GOT2)	1.1	1.2	0.004	0.15
CA345122	4-aminobutyrate aminotransferase, mitochondrial (GABT)	1.4	1.1	0.004	0.15
CA365793	Histone H3-K9 methyltransferase 4	1.3	1.2	0.006	0.15
***Protein folding/synthesis/breakdown (18.5%)***					
CA345680	Calpain-9	1.3	1.1	< 5.10^-4^	0.15
CA362332	Ubiquitin-conjugating enzyme E2 E1 (UB2E1)	1.3	1.4	< 10^-3^	0.1
CA351453	α-1,3-mannosyl-glycoprotein				
	2-beta-N-acetylglucosaminyltransferase	1.3	1.2	< 10^-3^	0.1
CA342952	Proteasome subunit beta type 7 precursor (PSB7)	1.6	1.5	0.002	0.1
BX302854	Proteasome-associated protein ECM29 homolog	1.5	1.6	0.003	0.1
***Xenobiotic and oxidant (22.2%)***					
CA359966	UDP-glucuronosyltransferase 2A1 precursor	1.3	1.6	< 5.10^-4^	0
BX302905	Metal-regulatory transcription factor 1	1.2	1.4	0.003	0.1
CA387417	Stress-activated protein kinase 3 (MK12)	1.5	1.3	0.004	0.15
BX078145	7,8-dihydro-8-oxoguanine triphosphatase	1.8	1.0	0.004	0.15
BX305962	ATP-binding cassette sub-family G member 2	1.5	1.6	0.007	0.1
BX299516	Cytochrome P450 2K4	1.3	1.2	0.007	0.15
***Purine and pyrimidine metabolism (15%)***					
BX295336	Nucleoside diphosphate kinase A	-1.3	-1.1	< 5.10^-4^	0.15
CA381176	Equilibrative nucleoside transporter 1	-1.4	-1.4	< 10^-3^	0.15
***Iron metabolism (3.7%)***					
BX909008	Transferrin receptor protein 1	-1.6	-1.4	0.005	0.15

1 According to both ANOVA (p < 0.01) and SAM (FDR < 0.15) analysis

#### Hepatic transcripts involved in a line per diet interaction

**Figure 1:**
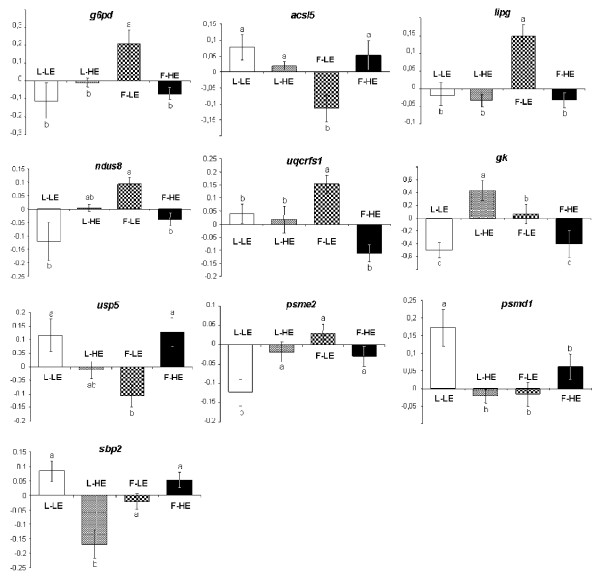
**Mean-centered, unit-normalized transcript levels of metabolism-related genes exhibiting significant line/diet interactions in the microarray experiment**. *g6pd*: glucose 6-phosphate dehydrogenase; *Acsl5*: long-chain acyl-CoA synthetase 5; *lipg*: endothelial lipase; *ndufs8*: NADH dehydrogenase [ubiquinone] iron-sulfur protein 8; *uqcrfs1*: cytochrome b-c1 complex subunit; *gk*: glucokinase; *usp5*: Ubiquitin carboxyl-terminal hydrolase 5; *psme2*: Proteasome activator complex subunit 2;*psmd1*: 26S proteasome non-ATPase regulatory subunit 1; *Sbp2*: selenium-binding protein 2. Data are presented as means of 6 samples ± SD. * P < 0.01.

Twenty-six transcripts for which the effects of the diet were dependent on genotype were detected by the two-way ANOVA. Two of these encoded for proteins involved in immune function, eight in cellular processes, and one in trafficking, four encoded for transcription factors, and ten were involved in metabolic pathways. The latter are represented in figure 1. It is of note that six of the ten significant interactions that concerned metabolic genes occurred in the F-LE group: transcript levels of four genes involved in lipid metabolism and energy production (glucose 6-phosphate dehydrogenase [*g6pd*], endothelial lipase [*lipg*], NADH dehydrogenase [ubiquinone] iron-sulfur protein 8 (*ndus8*), and cytochrome b-c1 complex subunit [*uqcrfs1*]) were increased in the F-LE group, whereas the expression of long-chain acyl-CoA synthetase 5 [*acsl5*], involved in the activation of long chain fatty acids, and ubiquitin carboxyl-terminal hydrolase [*usp5*], involved in proteolysis, were concomitantly decreased.

#### Confirmation of microarray data by real time RT-PCR

Changes in gene expression demonstrated by microarray analysis were further confirmed with a small set of genes using real time RT-PCR performed on nine trout liver samples per experimental group, including the samples we used in the microarray experiment (see additional file 3). Genes were selected for each category of effects we observed, i.e. *fads2 *(encoding for the protein Δ6-FAD, or delta-6-desaturase) and *pgdh3 *(D3-phosphoglycerate dehydrogenase), which exhibited a significant diet-induced change, *got2 *(aspartate aminotransferase) and *mdh *(malate dehydrogenase), that were found to be differentially expressed between lines, *pgd *(6-phosphogluconate dehydrogenase), that was regulated by both factors, and finally *g6pd *(glucose-6-phospahte dehydrogenase) and *gk *(glucokinase), for which a line/diet interaction was detected. The real time RT-PCR analysis of *fads2 *(delta-6-desaturase) not only confirmed the dietary effect detected by microarray analysis, but also revealed a significant line effect, with higher expression in the F line than in the L line.

### 2-D gel analysis of soluble liver proteins

**Figure 2:**
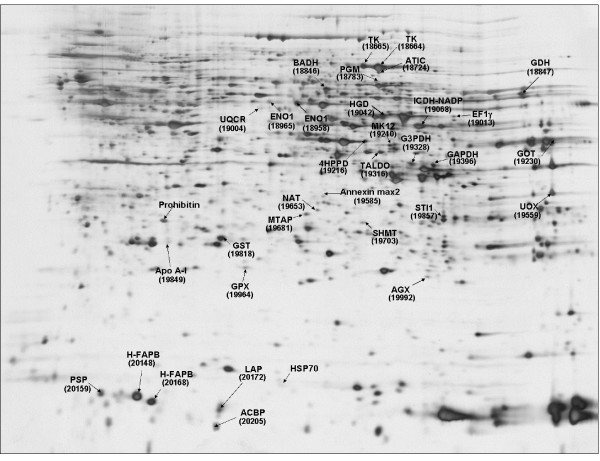
**Representative two-dimensional gel electrophoresis of rainbow trout liver proteins**. Total liver protein extract (150 μg) was separated first by IEF on 3–10 non-linear IPG drystrips, and then SDS-PAGE was performed on 12.5% polyacrylamide gel. The proteins were revealed by silver staining as described by Heukeshoiven and Dernick (1985). Differentially abundant proteins positively identified by trypsin digest fingerprinting are located by arrows, with their corresponding identity and spot ID.

Over 900 different spots were detected in at least one experimental condition, and 570 were detected in all four groups. According to the two-way ANOVA analysis, 265 protein spots showed a change in abundance between experimental conditions (p < 0.05). Of them, 70 were deemed to be of sufficient quality to allow peptide mass fingerprinting, and 36 yielded significant identities (see Additional file 4). Of the 36 proteins identified, 28 corresponded to proteins involved in metabolic pathways (78%). In particular, we identified two protein species of heart-type fatty acid binding protein (H-FABP), with different isoelectric points and molecular weights (figure 2). The best match for trypsin digest products from these two spots corresponded to the two EST printed on the microarray that we found to be differentially expressed using transcriptome analysis (Genbank accession numbers BX298066 and CU069693). We also identified two α-1 enolase and two transketolase protein species.

Three proteins involved in intracellular lipid transport (two H-FABPs and ACBP) were reduced with the HE diet, in agreement with the results observed with the transcriptome analysis. In addition, the two H-FABPs showed greater abundance in the F line than in the L line, an effect that was not evidenced at the transcript level. Glycerol-3-phosphate dehydrogenase (G3PDH) that is involved in phospholipid biosynthesis was also more highly expressed in the F line than in the L line. Finally, apolipoprotein A-1 (Apo A-1), the major protein in high-density plasma lipoprotein, was specifically up-regulated in the L line fed the HE diet (L-HE group).

Two proteins involved in the generation of precursor metabolites and energy were identified, i.e. NADP-dependent isocitrate dehydrogenase (ICDH-NADP), which was lower in fish fed the HE diet, and ubiquinol cytochrome c reductase, a component of the respiratory chain, that was more abundant in the F line than in the L line. ICDH-NADP was in addition markedly reduced in L fish fed the HE diet (L-HE group).

Six proteins involved in carbohydrate metabolism, particularly in glycolysis/gluconeogenesis (GAPDH, transaldolase, two α-1 enolase and two transketolase species) were expressed at lower levels in fish fed the HE diet compared to fish fed the LE diet. With regard to the selection effect, GAPDH, phosphoglucomutase (PGM), one α-enolase and one transketolase were more abundant in the F line than in the L line. In addition, line/diet interactions were observed for PGM, the two α-1 enolases and the two transketolases that were considerably less abundant in L fish fed the HE diet (L-HE group).

Several key enzymes involved in amino acid metabolism were identified: glutamate dehydrogenase (GDH) and alanine:glyoxylate aminotransferase (AGX), both involved in amino acid transamination, and betaine aldehyde dehydrogenase (BADH), involved in sulfur amino acid bioconversion pathways, were expressed at lower levels in fish fed the HE diet, whereas serine hydroxymethyl transferase was expressed at higher level in these fish. Aspartate aminotransferase (GOT), GDH, AGX and homogentisate 1, 2-dioxygenase (HGD) were detected in greater abundance in the F line than in the L line. In contrast, serine hydroxymethyltransferase (SHMT) and 4-hydroxyphenylpyruvate dioxygenase (4HPPD) were expressed at higher levels in the L line.

Finally, we identified four proteins related to oxidative cell status, i.e. glutathione peroxidase (GPX) and glutathione S transferase (GST), involved in the anti-oxidant process, as well as stress-activated protein kinase 3 (MK12) and arylamine N-acetyltransferase, that are induced by oxidative stress conditions. Lower levels of the two anti-oxidant enzymes were detected in the F line than in the L line, whereas the two stress-induced proteins were less abundant in the latter. Of these four proteins, only GPX showed a diet-induced change in abundance, with higher protein levels upon HE diet feeding.

## Discussion

### Agreement between transcriptome and proteome analyses

Transcriptome and proteome analysis constitute powerful tools for obtaining a view of the changes induced by genetic selection procedures and molecular adaptations to dietary treatments. The combination of the two approaches was justified in several respects. Indeed, the expression of a transcript and that of its corresponding protein are not necessarily related. Although much relevant information can be obtained from proteome analysis alone, the current proteomics technologies have some limitations of a technical order [21]. In addition, we are currently limited in the sequenced proteins available for fish species, in particular rainbow trout. About half of the spectra generated in this study led to significant protein identification. The combination of microarray and proteome analyses therefore makes it possible, to some extent, to get round the drawbacks associated with each method and to extract complementary information from these two independent methods.

In the present study, these two overall approaches provided consistent results. Some of the differentially expressed proteins we identified were also found on microarray to have altered mRNA levels (two H-FABPs, ACBP, GOT, GAPDH and the stress-activated protein kinase 3 [MK12]). The changes in abundance detected for these proteins were in accordance with the changes observed at the mRNA level.

### Long term effects of a high energy/high lipid diet on the hepatic gene and protein expression profiles

One of the aims of the present study was to examine the changes in hepatic gene and protein expression profiles induced by long term feeding of a high energy/high fat diet. As could be expected, given the large number of genes that are transcriptionally regulated by dietary fatty acids, particularly PUFAs [22], a high proportion of the transcripts that showed differential expression between dietary groups were involved in lipid metabolism. Three proteins involved in intracellular fatty acid transport (one ACBP and two H-FABPs) exhibited lower abundance at both mRNA and protein levels in fish fed the HE diet. H-FABPs belong to a family of small, cytosolic proteins that bind long-chain fatty acids and cholesterol. They are predominantly expressed in the heart, skeletal muscle and testes of mammals [23], but have been detected in a wider range of tissues, including the liver, in several fish species such as zebrafish, mummichog and lamprey [24-26]. Whereas H-FABP is thought to be involved in fatty acid uptake and transport toward mitochondrial β-oxidation in muscle tissues, as evidenced in mammals and Atlantic salmon [27-29], indications that H-FABP may also mediate intracellular fatty acid sequestration and transport toward the lipogenic process in the liver and oocytes have been reported in zebrafish [26]. Our results support the latter assumption, since the change in H-FABP level coincided with a decrease in the activity of key lipogenic enzymes [30]. ACBP is an intracellular lipid-binding protein that selectively binds medium and long chain acyl-CoA esters (C14–C22) with high specificity [31]. Studies in yeast [32] and the mouse [33] have suggested that ACBP may play a role in the synthesis of very long chain fatty acids, with dual regulation by both PPARα and SREBP-1, as for Δ5 and Δ6 desaturases [34]. The HE diet down-regulated the gene expression of 6-phosphogluconate dehydrogenase (*6-pgd*), a key enzyme of the pentose phosphate pathway that provides NADPH for the lipogenic process. The HE diet also decreased transcript level of Δ6-desaturase (Δ*6-fad*), a rate-limiting enzyme for the synthesis of highly unsaturated fatty acids (HUFAs). Fish oil is particularly rich in (n-3) polyunsaturated fatty acid and several studies performed in mammals [35] and salmonids [36-38] have demonstrated that a HUFA-rich diet reduces hepatic mRNA levels of Δ5- and Δ6-desaturases. Increasing the level of dietary (n-3) fatty acid supply has been shown to enhance enzymes of the antioxidant defense system such as GPX and catalase at both activity and mRNA levels [39]. Accordingly, we found that protein levels of GPX, a key enzyme in the antioxidant defense system, were increased in fish fed the HE diet, which contained 15% fish oil.

The protein abundance of GDH and AGX, two key enzymes of amino acid transamination and indicators of metabolic utilization of dietary amino acids, was lower in fish that received the HE diet. Some transcripts involved in proteasome-dependent proteolysis were also expressed at lower level with this diet. Fish swiftly use proteins as oxidative substrates [40,41]. Thus, increasing non-protein energy-yielding nutrients such as lipids generally leads to a protein sparing effect, probably by redirecting dietary protein and amino acids from energy production toward tissue deposition [42,43]. This is consistent with the enhanced protein efficiency ratio and growth performance observed in fish fed the HE diet, as previously described [30]. Feeding the HE diet was also associated with reduced levels of transcripts and proteins involved in sulfur amino acid bioconversion, such as 3-PGDH, CGL, or BADH. This might reflect a decrease in amino acid turnover in response to higher lipid supply, a nutritional context that would allow the cell to rely less heavily on the use of essential amino acids such as methionine to meet their energy requirements.

Long term feeding a diet supplemented in fish oil has been shown to induce substantial lowering of blood triglycerides (at least in part) by inhibiting the production and secretion of triglyceride-rich lipoprotein particles by the liver [44]. The plasma triglyceride level was consistently lower in fish fed the HE diet than in fish fed the LE diet. A reduction in lipogenesis associated to a stimulation of fatty acid oxidation in the liver, as suggested by our previous results [30], resulting from the higher level of fish oil provided by the HE diet might have decreased triglyceride and VLDL secretion in circulating blood.

### Hepatic transcripts and proteins affected by the selection procedure

In contrast to the results observed regarding the dietary effect on hepatic gene expression, the transcriptome analyses revealed minor changes in lipid metabolism induced by the selection procedure. However, the proteome approach yielded some complementary information, as higher levels of the two H-FABP proteins were present in the livers of the F line compared to the L line. This might have been due to post-transcriptional regulation, or to increased stability of these two proteins in the F line hepatocytes. Abundance of G3PDH, involved in phospholipids biosynthesis, was also increased in F fish livers. In addition, real time RT-PCR measurement of *fads2*, encoding for the delta-6-desaturase, an enzyme involved in fatty acid desaturation, showed increased mRNA levels in the F line. All these results, together with increased gene expression of 6-PGD, a key enzyme that provide NADPH for lipogenic process, suggest greater hepatic lipid biosynthesis in the F line. This is in good agreement with the higher activity of acetyl-CoA carboxylase, as previously evidenced [30], although no differences in fatty acid synthase activity could be found between the two lines [30].

However, most of the alterations induced by the selection procedure we observed occurred in protein and amino acid metabolism. In particular, levels of mitochondrial aspartate aminotransferase (GOT), GDH and AGX, three key enzymes that play a major role in amino acid catabolism, were increased in the livers of F fish. Transcripts and proteins that functioned in amino acid bioconversion (BHMT and GABT) and in proteasome-dependent proteolysis were also enhanced in this line. All these findings may reflect an increase in hepatic flux for energy production through amino acid metabolism in the F line compared to the L line. This is in agreement with the lower growth rate and protein efficiency observed for the F line [30]. Interestingly, some studies have reported strong evidence that amino acid catabolism may be negatively regulated by the peroxisome proliferator-activated receptor α (PPARα) in rodent liver [45-47]. In agreement with this, PPARα expression was lower in the F line than in the L line [30]. The increase in amino acid metabolism suggested here could thus be mediated through weaker inhibition of related genes by PPARα. This phenomenon has previously only been described in the mouse and rat. Although this remains to be confirmed by appropriate experiments in fish, the fact that such a mechanism might also operate in lower vertebrates would support the idea that regulation of amino acid metabolism by PPARα might be evolutionarily conserved throughout vertebrates.

The lower protein levels of two key enzymes in the antioxidant defense system (GPX and GST) observed in the F line might be explained by a lower production of reactive oxygen species (ROS) derived from fatty acid oxidation.

Taken together, all these findings suggest that a difference in nutrient utilization occurs between the two lines, the L line having a higher propensity to oxidize fatty acids than the F line [30], which may use comparatively more protein and amino acids for energy production. This difference in nutrient utilization could be at least in part orchestrated by PPARα, exerting opposing controls of fatty acid oxidation and amino-acid catabolism.

Plasma triglyceride levels were higher in the F line than in the L line. Increased fatty acid synthesis and decreased fatty acid oxidation in the liver of the F line might have led to enhanced fatty acid availability for triglyceride and subsequently VLDL production. Another hypothesis might be that a decreased extra-hepatic tissue lipid uptake in the F line compared to the L line also contribute to their higher blood triglyceride levels by lowering triglyceride clearance from plasma. Further analysis of the muscle and visceral adipose tissue lipid uptake should enlight this point.

### Comparing the effects of the HE diet and upward selection for muscle fat content on hepatic expression profiles

Significant effect exerted by both dietary treatment and genetic background were observed for some liver transcripts and proteins involved in processes such as cell cycle, transcription/translation, immune response and metabolic pathways. Overall, the HE diet and upward selection for muscle fat content exerted opposite effects in the liver. In particular, the expression of genes and proteins involved in lipid biosynthesis and amino acid and protein catabolism were decreased by the HE diet, and were increased in the F line compared to the L line. Some transcripts and proteins were involved in line/diet interactions, but they did not fall into function-related groups, except for the protein involved in glycolysis/neoglucogenesis, the abundance of which was decreased in the livers of fish from the L-HE group. Gene expression level of GK, a key enzyme of glycolysis that plays a major role in glucose homeostasis, was particularly high in the L-HE group compared to the three other groups. We are currently not able to explain the magnitude of changes observed for GK gene expression, since these changes are not correlated with glycaemia. This might reflect a disturbance of carbohydrate metabolism in the L line. Further analyses are needed, such as glucose tolerance test or post-prandial kinetics of glycaemia and GK gene expression. The relative few number of genes and proteins affected by both factors as well as line/diet interactions observed in the present work is in agreement with the results derived from our previous study [30]. All together, these results suggest that the dietary treatment and the genetic selection used in this study to manage muscle fattening are likely to act through different metabolic actors in the liver. The present study reveals that the cumulative effect exerted by the genetic selection and the high energy diet on muscle fattening is not associated with cumulative changes of hepatic metabolic pathways.

## Conclusion

The combination of liver transcriptome and proteome analysis led to the identification of several molecules that responded to the dietary treatments and the genetic selection for muscle fattening.

The increase in dietary energy and lipid supply provided by the HE diet induced significant changes in the hepatic transcriptome and proteome. The present findings confirmed the effects of long term feeding of a high-PUFA diet on the expression of genes and proteins involved in fatty acid desaturation and anti-oxidant metabolism previously described in mammals, suggesting that the underlying molecular mechanisms are evolutionarily conserved. The use of high throughput technologies led to the identification of previously unappreciated molecular actors, such as those involved in amino acid and protein metabolism, which responded to long term feeding with a high energy/high fat diet. They provide complementary information on the effects of dietary fat levels on genes involved in the regulation of energy metabolism.

Although muscle was the direct target, the selection procedure applied significantly affected hepatic metabolism. The main changes observed were in transcripts and proteins involved in amino acid and protein catabolism that were higher in the F line than in the L line. Some transcripts involved in lipogenesis were also increased in the F line compared to the L line. To our knowledge, the present study is the first that provides insights into the hepatic metabolic changes associated with differences in body fat distribution in a context of similar whole body fat content. We identified genes and proteins that could be used as markers to prevent excess muscle fat accumulation.

Further analysis of the muscle and visceral adipose tissue transcriptome and/or proteome will provide greater understanding of the mechanisms that are responsible for the differences in fat allocation between the two lines and of the fattening effects of a high energy/high fat diet on these body compartments.

## Methods

### Experimental animals and diets

The animals used in this study were the same as those described in Kolditz et al. [30]. Briefly, two lines of rainbow trout, a lean muscle line (L) and a fatty muscle line (F), were obtained after three generations of divergent selection for high or low muscle fat content, evaluated using a non-destructive method (Distell Fish Fatmeter) in live fish. Triplicate groups of fish of both lines were fed diets containing either 100 g (LE diet) or 230 g (HE diet) lipids/kg dry matter, from the first feeding for six months. Both diets were made from the same fishmeal-based mixture. About 15% fish oil was added to the HE diet to create a large difference in lipid content between the two diets. The inclusion of fish oil in the HE diet resulted in an increase in the overall energy content of the HE diet (+13%) compared to the LE diet. The main change in nutrient content involved the lipid fraction (+135% higher level in the HE diet) with an increase in n-3/n-6 polyunsaturated fatty acids ratio (+67% in the HE diet). Minor changes occurred in protein and starch content, both being decreased in the HE diet (-11% and -24%, respectively) as a consequence of a dilution effect (Table 6). At the end of the 6 month feeding trial, all fish were anesthetized with 2-phenoxyethanol at the recommended dose for surgical procedures (0.2 ml/l) 24 hours after the meal, and individually weighed. Nineteen fish per tank were sacrificed by a sharp blow on the head. Livers and viscera were weighed in order to calculate the hepato-somatic index (HSI (%) = [100 × (liver weight/body weight)]), and the viscero-somatic index (VSI (%) = [100 × (total viscera weight/body weight)]). Fillets from the left side of the fish were kept after trimming and skin removal as samples for analysis of lipid content. The livers of three fish per tank were sampled under RNAse-free conditions to perform gene and protein expression analysis. All the tissue samples were frozen in liquid nitrogen and stored at -80°C until analysis. The experiment was conducted according to the *National Guidelines for Animal Care of the French Ministry of Research*.

**Table 6:** Chemical composition of experimental diets

**Diets**	**LE**	**HE**
DM (%)	93.0	93.3
Protein (% DM)	57.6	51.1
Lipid (% DM)	9.8	23.1
n-3 FA/n-6 FA	1.5	2.5
Starch (% DM)	12.1	9.2
Energy (kJ/g DM)	21.0	23.8

DM, Dry Matter; FA, Fatty acids

### RNA extraction

The RNA extracts used for the present microarray and real time PCR analyses were the same as used by Kolditz et al. [30]. Total RNA was extracted from 9 individual livers per experimental condition using the TRIzol reagent method (Invitrogen, Carlsbad, CA, USA). Total RNA was quantified using spectrophotometry based on absorbance at 260 nm, and integrity was ascertained using the Agilent 2100 Bioanalyzer (Agilent Technologies, Kista, Sweden).

### cDNA microarray production

Nylon microarrays were obtained from the INRA-GADIE Biological Resources Center (Jouy-en-Josas, France) [48]. The microarrays contained 9023 distinct rainbow trout cDNAs originating from a normalized multi-tissue library [49]. Positive (luciferase) and negative (water) controls were also spotted on each microarray. This rainbow trout generic array was deposited in the Gene Expression Omnibus (GEO) database (Platform# GPL3650) [50].

### Microarray hybridization

Six hepatic RNA samples out of the 9 that were extracted from each experimental condition were randomly chosen and used for microarray hybridization at the INRA UMR1067 transcriptome facility (St-Pée-sur-Nivelle, France). A first hybridization was performed at 42°C for 48 h using a ^33^P-labelled oligonucleotide (TAATACGACTCACTATAGGG, sequence which is present at the extremity of each PCR product) to monitor the amount of cDNA in each spot. After stripping (3 hours 68°C, 0.1× SSC, 0.2% SDS), arrays were prehybridized for 1 h at 42°C in hybridization solution (5× Denhardt's, 5× SSC, 0.5% SDS). Labelled cDNAs were prepared from 3 μg of RNA by simultaneous reverse transcription and labelling for 1 hour at 42°C in the presence of 30 μCi [alpha-^33^P] dCTP, 0.6 μl 120 μM cold dCTP, 0.6 μl 20 mM dATP, dTTP, dGTP and 200 units SuperScript™ III Reverse Transcriptase (Invitrogen, Carlsbad, CA, USA) in 30 μL final volume. RNA was degraded by treatment at 68°C for 30 min with 1 μl 10% SDS, 1 μl 0.5 M EDTA and 3 μl 3 M NaOH, and then equilibrated at room temperature for 15 min. Neutralization was done by adding 10 μl 1 M Tris-HCl plus 3 μl 2N HCl. 2 μg of PolydA 80 mers were then added to the solution to saturate polyA tails. Arrays were incubated with the corresponding denatured labelled cDNAs for 48 h at 65°C in hybridization solution. After 3 washes (1 hours 68°C, 0.1× SSC 0.2% SDS), arrays were exposed 60 hours to phosphor-imaging plates before scanning using a FUJI BAS 5000.

### Microarray signal processing

Signal intensities were quantified using AGScan software [51], and normalization was performed using BASE software (BioArray Software Environnement), a MIAME-compliant database available at the bioinformatics facility SIGENAE [52]. Spots with an oligonucleotide signal lower than three times the background level were excluded from the analysis. After this filtering step, signal processing was performed using the vector oligonucleotide data to correct each spot signal according to the actual amount of DNA present in each spot. After correction, the signal was normalized by dividing each gene expression value by the median value of the array and then log transformed. Microarray data from this study have been deposited in the GEO database (Series# GSE12031) [50].

### Statistical analysis of microarray data

A total of 7740 clones out of 9023 (86%) passed through the background filter and were kept for further analysis. To evaluate potential interactions of diet and genotype, variations in gene expression were analyzed by two-way ANOVA (*p *< 0.01) for each gene, using Tiger TMEV 3.1 software [53], with dietary treatment and genotype as independent variables. When interactions were significant, means were compared using the Student Newman Keuls test. Analysis of the global effect of each mean factor (diet or genotype) was complemented by a two class unpaired comparison of Significance Analysis of Microarray (SAM) [54] using the Microsoft Excel software. The SAM analyses were performed on the whole set of data using different FDR cut offs (from 0 to 25% estimated false positives) on the following group comparisons: 1) HE vs LE, whatever the line 2) L vs F, whatever the diet. We chose a moderate FDR cut off of 0.15 that allow sufficient power while keeping the estimated number of false positives acceptable. Only transcripts concordantly identified by both statistical approaches were considered in the present study to assess the global effect of the diet on one hand, of the genotype on the other hand. Their expression ratios were calculated as 10^log(A)-log(B)^, A and B being respectively the mean expression value of HE and LE calculated within each line, or F and L, calculated within each dietary group.

### Data mining

Rainbow trout sequences originating from INRA AGENAE [55] and USDA [56] EST sequencing programs were used to generate publicly available contigs. The 8th version (Om.8, released January 2006) was used for BlastX [57] comparison against the Swiss-Prot database (January 2006) [58]. This was performed automatically for each EST spotted onto the membrane and used to annotate the 9023 clones of the microarray. For all genes identified as differentially abundant in the transcriptome analysis, ontologies were obtained using the GoMiner software [59] and complemented when necessary with information from the literature. When feasible, functional categories were allocated as they related to liver biology.

### Gene expression analysis: qRT-PCR

Nine individual samples per experimental condition were used as biological replicates, including the six samples used for microarray hybridization. Real-time PCR measurements were performed as described in Kolditz et al. [30] on a set of genes selected for each category of effects observed (dietary effect alone, selection effect alone, dietary effect + selection effect, line/diet interaction). The Genbank accession numbers, the sequences of the forward and reverse primers and the corresponding annealing temperature used for each gene tested for its expression are described in Additional file 5. Relative quantification of the target gene transcript with *ef1α *reference gene transcript [60] was made following the method described by Pfaffl [61] using the equation 1. The effect of dietary treatment, lines and line/diet interaction on real-time PCR data were tested using the statistical software SAS^® ^by means of a two-way ANOVA. Differences were considered significant when the probability level was < 0.05. When interactions were significant, means were compared using the Student-Newman-Keuls test.

### Protein extraction

Five individual liver samples for each experimental condition were used for proteome analyses. They were randomly chosen among the 6 animals per condition used for microarray analysis. Samples (100 mg) of frozen tissue were homogenized in lysis buffer (8 M urea, 4% (w/v) CHAPS, 40 mM Tris-HCl, 1 mM EDTA, 50 mM DTT, 1 mM AEBSF, 10 μM E-64) at room temperature, using an Ultra-Turrax homogenizer. Following homogenization, the tissue lysates were first centrifugated at 15,000 g for 30 min at 18°C, and subsequent supernatants were then centrifugated at 105,000 g for 1 hour at 18°C to remove any insoluble particles. The supernatant was then added to 1% (w/v) IPG buffer pH 3–10 NL (Amersham Biosciences) and stored at 70°C until gel electrophoresis was performed.

### Two-dimensional polyacrylamide gel electrophoresis

Gels were made in triplicate for each sample. Samples (150 μg total proteins) were diluted to 450 μL with DeStreak solution supplemented with 0.5% IPG buffer pH 3–10 NL (Amersham Biosciences). After incubation for 1 hour at room temperature, samples were loaded onto a 24 cm nonlinear immobiline dry strip, pH range 3–10 (IPG drystrips 3–10 NL, 24 cm; Amersham Biosciences). Isoelectric focusing (IEF) was performed using an IPGphor IEF system (Amersham Biosciences). After active in-gel sample rehydration, carried out at 30V over 12 hours, proteins were focused using five phases of stepped voltages from 200 to 8000 V, with total focusing of 78,810 Vh (all stages at 2 mA and 5 W). The strips were then equilibrated twice for 15 min with gentle shaking in equilibration solution containing 6 M urea, 50 mM Tris-HCl buffer, 30% glycerol, 2% SDS. DTT (65 mM) was added to the first equilibration solution in order to reduce disulfide bridges. Iodoacetamide (2.5%) and 0.5% bromophenol blue were added to the second solution. SDS-PAGE was then carried out using an Ettan Dalt6 unit (Amersham Biosciences). The IPG strip was laid onto a 12.5% constant concentration polyacrylamide slab gel (24 × 18 cm). Running was conducted using a two-step program, with 2.5 W/gel for 40 min, and then increased to 17 W/gel for 4 hours. For subsequent image analysis, fixed 2-D gels were silver-stained as described by Heukeshoven and Dernick [62]. Molecular masses of the proteins were determined by coelectrophoresis with standard protein markers. Isoelectric points were determined according to the IPG strip manufacturer's specifications.

### Analysis of 2D gels

The gels were scanned at a resolution of 200 dpi using an ImageScanner apparatus (GE Healthcare) and stored as TIF files. Subsequent analysis of the gel images was performed using the software package Image Master 2D Platinum Version 5.0 (Amersham Biosciences). Protein spots were detected using an automated procedure from the software combined with manual editing to remove artefacts. A reference gel was developed using 200 μg of total protein from a pool of all sample protein extracts in order to represent all the proteins they expressed, and against which all the remaining gels were matched using standard routines from the software. After the matching procedure, all protein spots were assigned a spot number from the reference gel. Individual protein spot abundance was determined by the area of the spot, multiplied by the density and referred to as the volume. The spot volumes were normalized to the total volume of all proteins detected on each gel. The normalized spot volume is described as the abundance of a particular protein spot. Data were subsequently analyzed using two-way ANOVA. The level of significance of difference was set at P < 0.05.

### Protein identification by peptide mass mapping

For protein identification, proteins resolved by 2D-PAGE were detected using silver staining compatible with protein mass spectrometry identification, as described by Yan et al. [63] and Coomassie Blue G250 [64]. Sample loading was increased from 150 μg to 600 μg. Proteins of interest were excised from the stained gel and subjected to in-gel trypsin digestion, as described by Com et al. [65]. The eluted peptides were subsequently analyzed by mass fingerprinting in a Matrix-assisted laser desorption time of flight (MALDI-TOF) mass spectrometer (Bruker Daltonics, Bremen, Germany). For protein identification, trypsin peptide masses were used to search the National Center for Biotechnology Information (NCBI) non-redundant sequences database using the MASCOT search program [66]. To utilize the EST nucleotide sequences now available for salmonid fish (108,000 sequences), the trypsin digest products were searched for in a database containing all fish cDNA sequences available (last update September 2006).

## Authors' contributions

CIK performed sample preparation, microarray hybridizations, bidimensional-electrophoresis, integrated the data and drafted the manuscript. GP contributed to the proteome analysis. MB performed quantitative RT-PCR. DE produced the microarrays. MS participated to statistical analysis. FL supervised the proteome analysis. FM supervised the whole study, conceived the experimental design, formulated diets and participated in writing the manuscript. All authors read and approved the final manuscript.

## Supplementary Material

Additional file 1 Transcripts exhibiting differential expression between the two dietary treatments (HE vs LE). The data provided represent the complete list of transcripts exhibiting differential expression between the two dietary treatments (HE vs LE) derived from each statistical method (ANOVA, p < 0.01 and SAM, FDR cut-off = 0,15). Click here for file

Additional file 2 Transcripts exhibiting differential expression between the two genetic lines (F vs L). The data provided represent the complete list of transcripts exhibiting differential expression between the two lines (F vs L) derived from each statistical method (ANOVA, p < 0.01 and SAM, FDR cut-off = 0,15). Click here for file

Additional file 3 Hepatic mRNA level of selected genes measured by RT-PCR (controls of the microarray data). mRNA levels of delta-6-desaturase, D-3-phosphoglycerate dehydrogenase, 6-phosphogluconate dehydrogenase, malate dehydrogenase, glucose 6-phosphate dehydrogenase and glucokinase in the liver of trout of both lines fed the diet LE or HE. Expression values (arbitrary units) are normalized with α-elongation factor 1 (EF1α)-expressed transcripts. Click here for file

Additional file 4 Hepatic proteins exhibiting differential expression between the experimental groups. The data provided represent the complete list of liver proteins exhibiting differential expression according to two-way ANOVA (p < 0.05), and positively identified by trypsin digest fingerprinting. Click here for file

Additional file 5 Accession numbers and primer sequences of genes selected for analysis by real time RT-PCR. Reverse and forward primer sequences and annealing temperature used to measure expression of 8 selected genes by real time RT-PCR. Click here for file
