# Supplementary material for: Changes induced by dietary energy intake and divergent selection for muscle fat content in rainbow trout (Oncorhynchus mykiss), assessed by transcriptome and proteome analysis of the liver
Source: BMC Genomics. 2008 Oct 29;9:506. doi: 10.1186/1471-2164-9-506 (PMC2612026; doi:10.1186/1471-2164-9-506)
Supplement: Additional file 3 — Hepatic mRNA level of selected genes measured by RT-PCR (controls of the microarray data). mRNA levels of delta-6-desaturase, D-3-phosphoglycerate dehydrogenase, 6-phosphogluconate dehydrogenase, malate dehydrogenase, glucose 6-phosphate dehydrogenase and glucokinase in the liver of trout of both lines fed the diet LE or HE. Expression values (arbitrary units) are normalized with α-elongation factor 1 (EF1α)-expressed transcripts. [file 1471-2164-9-506-S3.doc]

**Additional file 3.** Hepatic mRNA level of selected genes measured by RT-PCR (controls of the microarray data)

|  | **L line** | | **F line** | |  |  | P-values |  |  | HE/LE ratio | |  | F/L ratio | |
| --- | --- | --- | --- | --- | --- | --- | --- | --- | --- | --- | --- | --- | --- | --- |
| Diet | **LE** | **HE** | **LE** | **HE** |  | Diet | Line | Line*Diet |  | L line | F Line |  | LE diet | HE diet |
|  |  |  |  |  |  |  |  |  |  |  |  |  |  |  |
| ***fads2*** | 1.19±0.08 | 0.56±0.04 | 1.60±0.08 | 0.83±0.06 |  | *<10^-4^* | *<10^-4^* | 0.30 |  | -2.1 | -1.9 |  | 1.3 | 1.5 |
| ***pgdh3*** | 2.1±0.16 | 1.4±0.42 | 2.6±0.40 | 1.0 ±0.17 |  | *<10^-3^* | 0.88 | 0.10 |  | -1.5 | -2.6 |  | 1.2 | -1.4 |
| ***pgd*** | 0.93±0.12 | 0.58±0.03 | 1.65±0.23 | 0.82±0.13 |  | *<10^-4^* | *0.002* | 0.11 |  | -1.6 | -2.0 |  | 1.8 | 1.4 |
| ***mdh*** | 0.90±0.07 | 0.96±0.02 | 1.18±0.06 | 1.11±0.05 |  | 0.92 | *<10^-3^* | 0.27 |  | -1.1 | -1.1 |  | 1.3 | 1.2 |
| ***got2*** | 1.01±0.11^c^ | 0.98±0.06^c^ | 1.81±0.13^a^ | 1.28±0.15^b^ |  | *0.03* | *<10^-4^* | *0.04* |  | -1.0 | -1.4 |  | 1.8 | 1.3 |
| ***g6pd*** | 0.85±0.11^b^ | 0.59±0.04^c^ | 1.30±0.13^a^ | 0.54±0.03^c^ |  | *<10^-4^* | *0.04* | *0.01* |  | -1.4 | -2.4 |  | 1.5 | -1.1 |
| ***gk*** | 0.90±0.26^d^ | 14.72±5.25^a^ | 4.76±1.69^b^ | 3.08±0.98^c^ |  | 0.17 | 0.50 | *<0.01* |  | 16.3 | -1.5 |  | 5.3 | -4.8 |

mRNA was prepared from individual livers (n=9 per group), and included the 6 samples used for microarray analysis. Data, expressed in arbitrary units, are means ± SE of 9 samples performed in duplicate. ***fads2***, delta-6-desaturase; ***pgdh3***, D-3-phosphoglycerate dehydrogenase; ***pgd***, 6-phosphogluconate dehydrogenase; ***mdh***, malate dehydrogenase; *g6pd,* glucose 6-phosphate dehydrogenase**;** ***gk,*** glucokinase. Expression values are normalized with α-elongation factor 1 (EF1α)-expressed transcripts.  ^a, b, c, d^ P< 0.05. Means not sharing a common letter are significantly different from each other.
