# Supplementary material for: Changes induced by dietary energy intake and divergent selection for muscle fat content in rainbow trout (Oncorhynchus mykiss), assessed by transcriptome and proteome analysis of the liver
Source: BMC Genomics. 2008 Oct 29;9:506. doi: 10.1186/1471-2164-9-506 (PMC2612026; doi:10.1186/1471-2164-9-506)
Supplement: Additional file 4 — Hepatic proteins exhibiting differential expression between the experimental groups. The data provided represent the complete list of liver proteins exhibiting differential expression according to two-way ANOVA (p < 0.05), and positively identified by trypsin digest fingerprinting. [file 1471-2164-9-506-S4.doc]

**Additional file 4.** Hepatic proteins exhibiting differential expression between the experimental groups^1^

| ***Biological process*** | | Highest | L line | | F line | | p-value | | |
| --- | --- | --- | --- | --- | --- | --- | --- | --- | --- |
| Spot ID | Homology (*species*) | mascot score | LE diet | HE diet | LE diet | HE diet | Diet | Line | Line*Diet |
| ***Lipid metabolism*** | |  |  |  |  |  |  |  |  |
| 20148 | Heart-type fatty acid binding protein (H-FABP) *(O.mykiss)* | 98/69 | 0.771 ± 0.048 | 0.597 ± 0.045 | 0.879 ± 0.053 | 0.690 ± 0.054 | *<10^-3^* | *0.049* | 0.88 |
| 20168 | Heart-type fatty acid binding protein (H-FABP) *(O.mykiss)* | 81/69 | 0.294 ± 0.044 | 0.200 ± 0.011 | 0.456 ± 0.031 | 0.268 ± 0.027 | *<10^-4^* | *<10^-3^* | 0.13 |
| 20205 | Acetyl coenzyme A binding protein (ACBP) (*O. mykiss*) | 80/69 | 0.179 ± 0.018 | 0.136 ± 0.011 | 0.176 ± 0.017 | 0.101 ± 0.022 | *10^-3^* | 0.26 | 0.35 |
| 19849 | Apolipoprotein A-I (Apo A-I) *(O. Mykiss)* | 111/69 | 0.129 ±0.020 | 0.182 ± 0.018^a^ | 0.144 ±0.023 | 0.101 ± 0.013 | 0.80 | 0.095 | *0.016* |
| 19328 | Glycerol-3-phosphate dehydrogenase (G3PDH) (*S. Salar*) | 133/69 | 0.178 ± 0.013^b^ | 0.164 ± 0.011^b^ | 0.209 ± 0.013^b^ | 0.208 ± 0.027^b^ | 0.66 | *0.024* | 0.69 |
| ***Generation of metabolite precursors and energy*** | |  |  |  |  |  |  |  |  |
| 19068 | Isocitrate dehydrogenase, NADP-dependant (ICDH-NADP) | 78/78 | 0.294 ± 0.027^a^ | 0.098 ± 0.018^c^ | 0.259 ± 0.022^a^ | 0.164 ±0.026^b^ | *<10^-6^* | 0.51 | *0.034* |
| 19004 | Ubiquinol cytochrome c reductase (UQCR) *(O. mykiss)* | 80/78 | 0.021 ± 0.003 | 0.020 ± 0.003 | 0.027 ± 0.002 | 0.028 ± 0.003 | 0.92 | *0.016* | 0.68 |
| ***Carbohydrate metabolism*** | |  |  |  |  |  |  |  |  |
| 19396 | Glyceraldehyde-3-phosphate dehydrogenase (GAPDH) *(S.trutta)* | 103/69 | 0.183 ± 0.023 | 0.089 ± 0.012 | 0.237 ± 0.020 | 0.147 ± 0.011 | *<10^-5^* | *0.004* | 0.92 |
| 18965 | Alpha-1 enolase-1 (ENO1) *(S. trutta)* | 92/78 | 0.206 ± 0.015^a^ | 0.119 ± 0.008^b^ | 0.193 ± 0.009^a^ | 0.188 ± 0.018^a^ | *0.031* | 0.22 | *0.028* |
| 18958 | Alpha-1 enolase-1 (ENO1) *(S. trutta)* | 92/78 | 0.094 ± 0.007^a^ | 0.062 ± 0.007^b^ | 0.087 ± 0.006^a^ | 0.087 ± 0.010^a^ | *<10^-3^* | *0.033* | *0.003* |
| 19316 | Transaldolase (TALDO) (*H. sapiens*) | 130/69 | 0.127 ± 0.013 | 0.086 ± 0.011 | 0.092 ± 0.009 | 0.098 ± 0.012 | *0.014* | 0.63 | 0.70 |
| 18783 | Phosphoglucomutase (PGM) (*O. cuniculus*) | 80/69 | 0.033 ± 0.002^a^ | 0.021 ± 0.004^b^ | 0.032 ± 0.004^a^ | 0.038 ± 0.004^a^ | 0.39 | *0.019* | *0.013* |
| 18664 | Transketolase (TK) | 75/69 | 0.364 ± 0.040^a^ | 0.180 ± 0.022^b^ | 0.361 ± 0.034^a^ | 0.327 ± 0.032^a^ | *0.002* | *0.03* | *0.026* |
| 18665 | Transketolase (TK) | 82/69 | 0.193 ± 0.021^a^ | 0.089 ± 0.016^b^ | 0.181 ± 0.021^a^ | 0.163 ± 0.017^a^ | *0.003* | 0.11 | *0.028* |
| ***Amino-acid derivative metabolism*** | |  |  |  |  |  |  |  |  |
| 19230 | Aspartate aminotransferase, mitochondrial precursor (GOT2) (*R. norvegicus*) | 102/69 | 0.109 ± 0.017 | 0.074 ± 0.014 | 0.172 ± 0.020 | 0.173 ± 0.025 | 0.38 | *10^-4^* | 0.37 |
| 19200 | Alanine:glyoxylate aminotransferase (AGX) *(S. salar)* | 120/69 | 0.206 ± 0.024 | 0.151 ± 0.022 | 0.255 ± 0.024 | 0.243 ± 0.020 | *0.015* | *0.048* | 0.051 |
| 18847 | Glutamate dehydrogenase (GDH) *(S. salar)* | 137/78 | 0.95 ± 0.014 | 0.038 ± 0.009 | 0.123 ± 0.020 | 0.074 ± 0.008 | *0.004* | *0.017* | 0.46 |
| 18846 | Betaine aldehyde dehydrogenase (BADH) *(G.callarias)* | 143/69 | 0.145 ± 0.012^a^ | 0.089 ± 0.009^d^ | 0.140 ± 0.007^b^ | 0.125 ± 0.011^c^ | *0.003* | *0.041* | *<10^-3^* |
| 19042 | Homogentisate 1 2-dioxygenase (HGD) (*M. musculus*) | 112/69 | 0.180 ± 0.014 | 0.141 ± 0.009 | 0.229 ± 0.018 | 0.220 ± 0.022 | 0.13 | *<10^-3^* | 0.34 |
| 19703 | Serine hydroxymethyltransferase (SHMT) (*H. sapiens*) | 72/69 | 0.045 ± 0.005 | 0.170 ± 0.013^a^ | 0.041 ± 0.003 | 0.046 ± 0.006 | <10^-10^ | *<10^-9^* | *<10^-9^* |
| 19216 | 4-hydroxyphenylpyruvate dioxygenase (4HPPD) | 101/69 | 0.145 ± 0.012 | 0.156 ± 0.006 | 0.115 ± 0.009 | 0.116 ± 0.015 | 0.59 | 0.002 | 0.62 |

**Additional file 4.** (continued)

| ***Biological process*** | | Highest | L line | | F line | | p-value | | |
| --- | --- | --- | --- | --- | --- | --- | --- | --- | --- |
| Spot ID | Homology (*species*) | mascot score | LE diet | HE diet | LE diet | HE diet | Diet | Line | Line*Diet |
| ***Purine and pyrimidine metabolism*** | |  |  |  |  |  |  |  |  |
| 19559 | Urate oxidase (UOX) *(S. Salar)* | 101/78 | 0.133 ± 0.017 | 0.107 ± 0.014 | 0.178 ± 0.014 | 0.143 ± 0.014 | *0.049* | *0.01* | 0.74 |
| 18724 | 5-Imidazole-4-carboxamide ribonucleotide formyltransferase/IMP cyclohydrolase (ATIC) *(S. salar)* | 77/69 | 0.039 ± 0.005^a^ | 0.022 ± 0.004^b^ | 0.033 ± 0.004^a^ | 0.039 ± 0.005^a^ | 0.24 | 0.22 | *0.014* |
| 19681 | 5’ –methylthioadenosine phosphorylase (MTAP) *(M. musculus)* | 86/69 | 0.075 ± 0.004 | 0.076 ± 0.004 | 0.062 ± 0.004 | 0.063 ± 0.005 | 0.80 | *0.003* | 0.98 |
| ***Xenobiotic and oxidant metabolism*** | |  |  |  |  |  |  |  |  |
| 19964 | Glutathione peroxidase (GPX) (*R. norvegicus*) | 90/69 | 0.051 ± 0.003 | 0.080 ± 0.008 | 0.041 ± 0.003 | 0.064 ± 0.007 | *<10^-4^* | *0.019* | 0.61 |
| 19818 | Glutathione S transferase (GST) (*O. mykiss*) | 112/69 | 0.284 ± 0.019 | 0.258 ± 0.011 | 0.226 ± 0.016 | 0.226 ± 0.016 | 0.11 | *0.04* | 0.12 |
| 19240 | Stress-activated protein kinase 3 (MK12) (*H. Sapiens*) | 98/78 | 0.052 ± 0.006 | 0.042 ± 0.04 | 0.070 ± 0.006 | 0.058 ± 0.006 | *0.003* | 0.87 | 0.05 |
| 19653 | Arylamine N-acetyltransferase (NAT) *(G. gallus)* | 77/69 | 0.037 ± 0.003 | 0.034 ± 0.003 | 0.051 ± 0.004 | 0.047 ± 0.003 | 0.30 | *<10^-4^* | 0.85 |
| ***Transcription/translation*** | |  |  |  |  |  |  |  |  |
| 20159 | 14.3kDa perchloric acid soluble protein (PSP) | 83/69 | 0.179 ± 0.011^b^ | 0.231 ± 0.019^a^ | 0.241 ± 0.015^a^ | 0.216 ± 0.017^a^ | 0.39 | 0.15 | *0.02* |
| 19013 | Elongation factor 1 gamma (EF1γ) *(C. auratus)* | 83/69 | 0.023 ± 0.003^a^ | 0.014 ± 0.001^b^ | 0.021 ± 0.003^a^ | 0.024 ± 0.002^a^ | 0.21 | 0.16 | *0.03* |
| ***Miscellaneous*** | |  |  |  |  |  |  |  |  |
| 19585 | Annexin max2 *(O. latipes)* | 206/69 | 0.077 ± 0.011^a^ | 0.038 ± 0.004^b^ | 0.074 ± 0.013^a^ | 0.090 ± 0.014^a^ | 0.31 | *0.03* | *0.02* |
| 20051 | Prostaglandin dehydrogenase (PDH) *(P.hymadrias)* | 149/69 | 0.036 ± 0.004 | 0.048 ± 0.003 | 0.034 ± 0.003 | 0.038 ± 0.004 | *0.031* | 0.14 | 0.28 |
| 19309 | Phosphotriesterase_related protein (Parathion hydrolase-related protein) (*M.musculus*) | 104/69 | 0.055 ± 0.007^b^ | 0.104 ± 0.008^a^ | 0.052 ± 0.003^b^ | 0.053 ± 0.007^b^ | *<10^-3^* | *<10^-3^* | *<10^-^* |
| 19704 | Prohibitin (*D. rerio*) | 103/78 | 0.164 ± 0.010 | 0.119 ± 0.011 | 0.174 ± 0.010 | 0.155 ± 0.011 | *0.003* | *0.035* | 0.19 |

1According to two-way ANOVA (p<0.05), and positively identified by trypsin digest fingerprinting. .Spot ID refers to the number assigned to the spot in the reference gel. Data are means± SE of 5 samples performed in triplicate. ^a, b, c, d^ P< 0.05. Means not sharing a common letter are significantly different from each other.
