# Supplementary material for: Changes induced by dietary energy intake and divergent selection for muscle fat content in rainbow trout (Oncorhynchus mykiss), assessed by transcriptome and proteome analysis of the liver
Source: BMC Genomics. 2008 Oct 29;9:506. doi: 10.1186/1471-2164-9-506 (PMC2612026; doi:10.1186/1471-2164-9-506)
Supplement: Additional file 5 — Accession numbers and primer sequences of genes selected for analysis by real time RT-PCR. Reverse and forward primer sequences and annealing temperature used to measure expression of 8 selected genes by real time RT-PCR. [file 1471-2164-9-506-S5.doc]

**Additional file 5**. Accession numbers and primer sequences of genes selected for analysis by real time RT-PCR

| **Target gene** | **Abbreviated name** | **Databank** | **Accession number** | **Forward primer** | **Reverse primer** | **Annealing temperature (C°)** | **Amplicon size (pb)** |
| --- | --- | --- | --- | --- | --- | --- | --- |
| Δ-6 fatty acid desaturase | *Δ6-fad* | Genbank | CA371783 | *agggtgcctctgctaactgg* | *tggtgttggtgatggtaggg* | *59* | *175* |
| Glucose-6-phosphate dehydrogenase - | *g6pd* | Genbank | CA351434 | *ctcatggtcctcaggtttg* | *agagagcatctggagcaagt* | 59 | *176* |
| 6-phosphogluconate dehydrogenase | *6-pgd* | Genbank | CA342644 | *gtgccgtctgtgtctctga* | *ctgtgactgggttggtgatg* | 55 | *234* |
| Malate dehydrogenase | *mdh* | Genbank | CA351158 | *cccactcctcagagaggtca* | *tggctgcgatcagacagttg* | *57* | *239* |
| D-3-phosphoglycerate dehydrogenase | *3-pgdh* | Genbank | BX085755 | *agggcttggttggagcagtg* | *ttgaccaggttggggcaact* | *60* | *250* |
| Aspartate aminotransferase | *got* | Genbank | [BX076291](http://srs.ebi.ac.uk/cgi-bin/wgetz?-id+sessionid+-e+%5BEMBL-id:BX076291%5D+-view+EmblEntry) | *ttgtgtccgcaaggcagagg* | *cagagcgcaggacttggtga* | *60* | *100* |
| Hexokinase IV (Glucokinase) | *gk* | Genbank | BX860818 | *gaaggtgaaacccagaggaagc* | *tgaaggatcagaggtgggtgatt* | *59* | *253* |
| Elongation factor I alpha | *ef1α* | Genbank | AF498320 | *tcctcttggtcgtttcgctg* | *acccgagggacatcctgtg* | *59* | *159* |
